# Dysphagia and Muscle Weakness Secondary to Botulinum Toxin Type A Treatment of Cervical Dystonia: A Drug Class Analysis of Prescribing Information

**DOI:** 10.3390/toxins16100442

**Published:** 2024-10-15

**Authors:** Khashayar Dashtipour, Han S. Lee, Aaron Ellenbogen, Rashid Kazerooni, Todd M. Gross, David A. Hollander, Conor J. Gallagher

**Affiliations:** 1 Department of Neurology/Movement Disorders, Loma Linda University, Loma Linda, CA 92354, USA; 2 Department of Neurology, The Permanente Medical Group, Greater Southern Alameda Area—San Leandro/Fremont, San Leandro, CA 94577, USA; han.neurology@gmail.com; 3 Quest Research Institute, Farmington Hills, MI 48334, USA; aellenbogen@comcast.net; 4 Revance Therapeutics, Nashville, TN 37203, USA; rashid.kazerooni@revance.com (R.K.); tgross@revance.com (T.M.G.); david.hollander@revance.com (D.A.H.); cgallagher@revance.com (C.J.G.)

**Keywords:** botulinum toxins, type A, cervical dystonia, dysphagia, dystonic disorders, movement disorders, muscle weakness, neuromodulator, peptides

## Abstract

The first-line management of cervical dystonia (CD) symptoms is intramuscular injection of botulinum toxin type A (BoNTA). However, a comparison of safety among BoNTAs is difficult because, per regulatory authorities, units of BoNTA activity are not interchangeable. Dysphagia and muscle weakness are widely considered two key adverse events to monitor closely in the treatment of CD. This integrated analysis compared the safety of BoNTAs approved for CD in the US by evaluating relationships between the incidence of dysphagia and muscle weakness in prescribing information and the core neurotoxin content. Coefficients The coefficients of determination (R^2^) and trendlines were estimated via regression-based lines of best fit. Adverse drug reaction (ADR) rates were strongly correlated with core neurotoxin amounts for conventional BoNTAs (slope coefficients: dysphagia = 0.048, R^2^ = 0.74; muscle weakness = 0.096, R^2^ = 0.82). The published ADR rates at approved doses for conventional BoNTAs were higher compared with DaxibotulinumtoxinA (DAXI; DAXXIFY^®^, Revance Therapeutics, Inc., Nashville, TN, USA) by core neurotoxin content. The use of a core neurotoxin amount was found to be an effective method for comparing the safety of BoNTA products. Current clinical trials suggest that DAXI, a novel BoNTA formulation, provides a potentially wider safety margin compared with other approved BoNTAs for CD. The lower amount of core neurotoxin administered at approved doses compared with conventional BoNTAs may explain low on-target ADRs like muscle weakness, whereas reduced diffusion from the injection site is thought to be responsible for low off-target ADRs like dysphagia.

## 1. Introduction

Cervical dystonia (CD), the most common form of focal dystonia encountered in movement disorder clinics, is a clinical condition requiring lifelong treatment [1]. CD is characterized by involuntary movements and posturing of the head, neck, and shoulders that can be painful and, in some cases, might affect quality of life and activities of daily living [2,3]. First-line pharmacotherapy for managing CD symptoms is focal intramuscular injections of botulinum toxin type A (BoNTA), a highly potent neuromuscular blocking agent [4,5,6]. One of the common reasons for treatment adjustment or discontinuation is the emergence of adverse events, which are typically dose-dependent and can be the result of diffusion and the spreading of neurotoxin to adjacent non-target tissues [4,7,8,9]. Dysphagia and muscle weakness are common and of major concern when treating cervical dystonia with BoNTAs [4] because these events may limit the range of muscles selected for treatment (e.g., deeper muscles) [10]. Additionally, these concerns may affect dosing decisions and, thus, the optimal management of patients’ symptoms [10].

The safety margin for any pharmacotherapy can be defined as the gap between the efficacious dose and the dose at which adverse effects occur [11]. Wider safety margins may permit expanded muscle selection and/or allow for drug titration to optimize efficacy with a lower risk of adverse events. Preclinical studies have identified differences in the safety margins for some of the currently available BoNTA products [12]. Although these products have similar degrees and durations of clinical benefit, the significant differences in their adverse event profiles suggest that there is a correlation between preclinical observations and clinical practice [13,14,15]. This raises the possibility that the different BoNTA formulations might have different safety margins [12,16].

Commercial BoNTA preparations are formulated biologics containing a 150 kDa core neurotoxin as the active pharmaceutical ingredient [8]. The three conventional BoNTA products that are approved in the United States for use in patients with CD are onabotulinumtoxinA (Botox^®^, AbbVie, Dublin, Ireland) [13], abobotulinumtoxinA (Dysport^®^, Ipsen Biopharm Ltd., Wrexham, UK) [14], and incobotulinumtoxinA (Xeomin^®^, Merz Pharmaceuticals GmbH, Frankfurt, Germany) [15]. For onabotulinumtoxinA and abobotulinumtoxinA, the core neurotoxin is assembled in a multimeric complex with a non-hemagglutinin protein and varying amounts of hemagglutinin proteins that form a complex of variable size (900 kDa to 400 kDa), whereas incobotulinumtoxinA is manufactured to be free of these “accessory proteins” [8]. These conventional BoNTA products tend to have relatively similar clinical profiles and are formulated using the common excipient human serum albumin (HSA) in addition to a salt or sugar [8,13,14,15].

A novel BoNTA formulation, DaxibotulinumtoxinA, referred to herein as “DAXI” for simplicity (sold under the brand name and registered trademark DAXXIFY^®^ by Revance Therapeutics, Inc., Nashville, TN, USA), was approved for the treatment of CD by the U.S. Food and Drug Administration (FDA) in August 2023 [17]. DAXI, the first BoNTA formulated with a custom-engineered 35-amino acid (5 kDa) peptide (RTP004), is manufactured without unnecessary accessory proteins and does not contain HSA [17,18]. The RTP004 peptide helps reduce the tendency of the protein to form aggregates and to adsorb to the glass of the vial (roles typically achieved with HSA in conventional BoNTA formulations [8]). Furthermore, the amino acid composition of RTP004 renders it highly positively charged, enabling it to bind to negatively charged regions of the 150 kDa core neurotoxin [19,20]. The peptide has been reported to improve clinical performance via multiple mechanisms, including enhanced neuronal cell binding affinity leading to increased SNAP-25 cleavage and reduced diffusion from the injection site [20,21]. These mechanistic enhancements may play a role in increasing the neuronal bioavailability of the DAXI formulation, which has demonstrated a long duration of effect in several well-controlled studies [22] despite the low amounts of core neurotoxin being administered. The tight localization of neurotoxin to the injected area, due to enhanced neuronal binding affinity and increased SNAP-25 cleavage, might explain the reduced diffusion and spread of DAXI from the injection site and potentially contribute to the favorable adverse event profile [22].

One of the challenges that clinicians face when comparing BoNTA products, or when switching patients from one product to another, is that BoNTA products are typically dosed in units of biological activity, with a unit being defined as the mass (usually in picograms) of BoNTA that produces a median lethal dose (LD50) in a mouse bioassay [23]. The units of biological activity of DAXI and all BoNTA products are specific to each product and the method used to determine potency, and the FDA mandates label language stating that units are not interchangeable across BoNTA products [13,14,15,17]. Notwithstanding this, the quantification of the mass of the core 150 kDa neurotoxin per dose might enable a comparison of biological activity across BoNTAs. However, the relationship of core neurotoxin content with the adverse event profiles of BoNTA products has not been explored. The aim of this analysis was to compare the safety of the four BoNTA products approved by the FDA for CD by evaluating safety data from prescribing information (PI) and the reported core neurotoxin content [13,14,15,17].

## 2. Results

**Figure 1:**
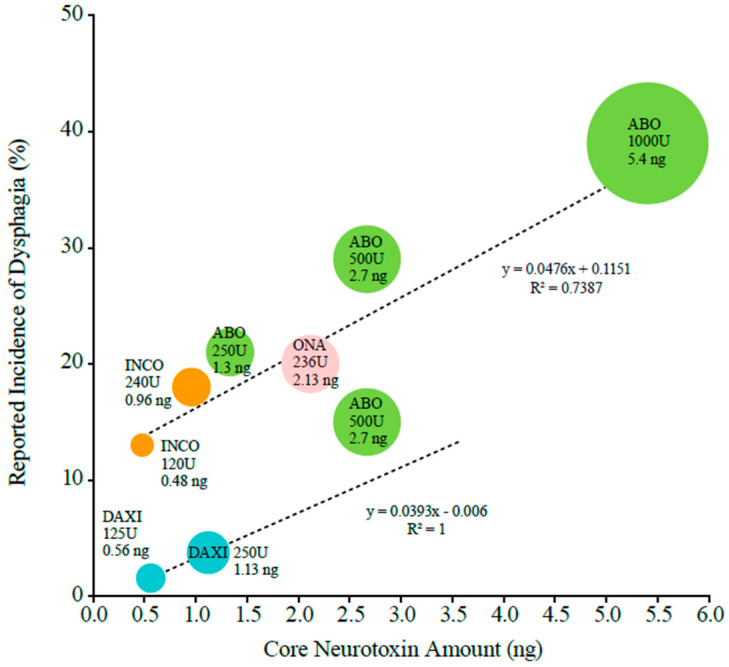
The relationship between the incidence of dysphagia reported in prescribing information for cervical dystonia and the core botulinum toxin A neurotoxin amount. The slope of the line is shown, where y is the reported incidence, x is the core neurotoxin amount, 0.1151 is the y intercept, and 0.0476 is the slope of the line. The circle size is representative of nanogram amounts of neurotoxin at each approved dose. Abbreviations: ABO—abobotulinumtoxinA; DAXI—DaxibotulinumtoxinA; INCO—incobotulinumtoxinA; ONA—onabotulinumtoxinA.

The incidence of dysphagia was positively correlated with core neurotoxin amounts for conventional BoNTAs (slope coefficient = 0.0476, R^2^ = 0.74). The slope coefficient from the line of best fit translates to an increase of 4.8% in the incidence of dysphagia per additional nanogram of core neurotoxin (Figure 1). For conventional BoNTAs, the incidence of dysphagia ranged from 13% for incobotulinumtoxinA at 0.48 ng of core neurotoxin (120 U) to 39% for abobotulinumtoxinA at 5.4 ng of core neurotoxin (1000 U). The published incidences for conventional BoNTAs were higher than those reported for DAXI when analyzed by core neurotoxin content (Figure 1). For DAXI, the incidence of dysphagia was 2% at 0.56 ng of core neurotoxin (125 U) and 4% at 1.13 ng of core neurotoxin (250 U), and these values did not fall along the line of best fit for conventional BoNTAs.

**Figure 2:**
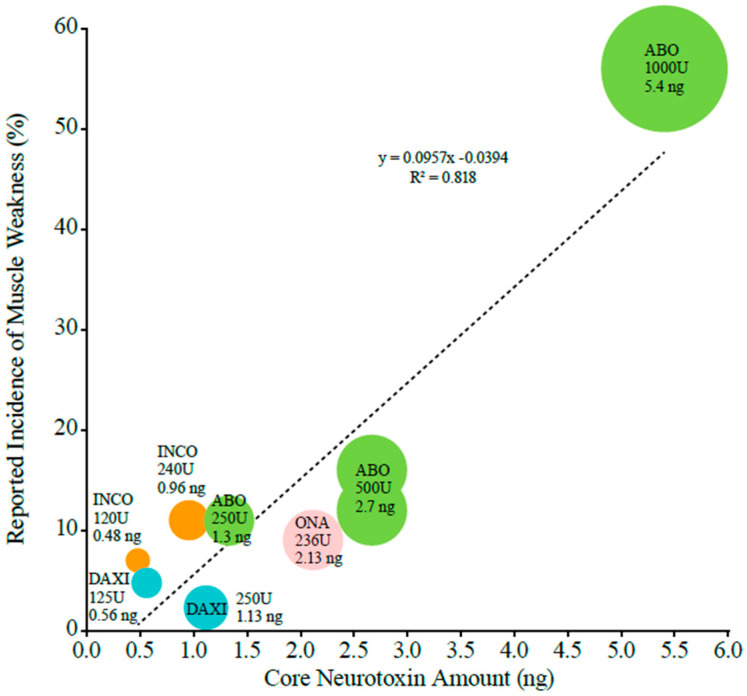
The relationship between the incidence of muscle weakness reported in prescribing information for cervical dystonia and the core botulinum toxin A neurotoxin amount. The slope of the line is shown, where y is the reported incidence, x is the core neurotoxin amount, 0.0394 is the y intercept, and 0.0957 is the slope of the line. The circle size is representative of nanogram amounts of neurotoxin at each approved dose. Abbreviations: ABO—abobotulinumtoxinA; DAXI—DaxibotulinumtoxinA; INCO—incobotulinumtoxinA; ONA—onabotulinumtoxinA.

The incidence of muscle weakness was also positively correlated with core neurotoxin amounts for conventional BoNTAs (slope coefficient = 0.0957, R^2^ = 0.82). The slope of the line of best fit translates to an increase of 9.6% in the incidence of muscle weakness per additional ng of core neurotoxin (Figure 2). For conventional BoNTAs, the incidence of muscle weakness ranged from 7% for incobotulinumtoxinA at 0.48 ng of core neurotoxin (120 U) to 56% for abobotulinumtoxinA at 5.4 ng of core neurotoxin (1000 U). Similar to dysphagia, the published incidences for conventional BoNTAs were higher than those reported for DAXI when analyzed by core neurotoxin content (Figure 2). For DAXI, the incidence of muscle weakness was 5% at 0.56 ng of core neurotoxin (125 U) and 2% at 1.13 ng of core neurotoxin (250 U), and these values appeared to fall along the line of best fit for conventional BoNTAs.

## 3. Discussion

This analysis has demonstrated for the first time a relationship between the core neurotoxin amount and key adverse events for the use of conventional BoNTAs in the treatment of CD. The R^2^ values for dysphagia and muscle weakness were ≥0.70, suggesting that a large proportion of these adverse event rates could be explained by the amount of core neurotoxin present in each dose. While there is no universal consensus on what is an acceptable cutoff for R^2^, values greater than 0.70 are generally considered to represent a strong correlation in the medical literature [24,25]. Thus, these data suggest that the relationship between the core neurotoxin amount and adverse event rates may serve as a more useful method than units of activity for comparing adverse events with BoNTA treatment.

The two key adverse events in this analysis are representative of the different effects of BoNTA products. Dysphagia is often considered to be an off-target effect resulting from the local spread of neurotoxins to adjacent muscles of deglutition [26]. In contrast, muscle weakness generally results from the over-weakening of injected muscles or muscle combinations and is often considered an on-target effect [27]. For dysphagia, the DAXI data points did not fall along the line of best fit that was determined for the conventional BoNTA products. Although pivotal trial data are available for only two DAXI doses, the DAXI regression line ran parallel and lower and did not intersect with that of the conventional BoNTA products. This suggests that there is a difference in the off-target tissue activity of DAXI relative to the other BoNTA products, which may be driven by the unique characteristics of the DAXI formulation. Findings from a mouse model study found that at a 1:1 unit dose ratio of DAXI to onabotulinumtoxinA, lower levels of diffusion to an adjacent muscle were seen for DAXI compared with onabotulinumtoxinA [28]. In fact, 2.5 times more units of DAXI than onabotulinumtoxinA was needed before a comparable spread to an adjacent muscle was observed. In the current analysis of clinical trials for cervical dystonia, when the products were compared unit for unit in the retrospective analysis, onabotulinumtoxinA demonstrated significantly greater spread into adjacent off-target muscle tissue compared with DAXI. Conversely, for muscle weakness, which is an on-target effect, the DAXI data points did fall along the line of best fit that was determined for the conventional BoNTA products. This suggests that, from a pharmacological perspective, DAXI behaves in a similar way to other BoNTA products in the target muscle. However, the amount of core neurotoxin that is required to achieve the desired clinical effect is lower for DAXI than for other BoNTA products, which may help explain the low rate of muscle weakness. This efficiency, in terms of core neurotoxin use, stems from DAXI’s novel peptide formulation, which results in greater bioavailability of the neurotoxin [21].

The safety of BoNTA products may be related to the components of the formulation that determine the efficiency of the delivery of toxin to nerve terminals (i.e., cell membrane binding, internalization, and the delivery of the light chain into the target neuron) [29,30]. Because DAXI has an efficient formulation that is mechanistically enhanced via a custom-engineered peptide, lower amounts of neurotoxin can be administered, which may reduce off-target adverse events related to diffusion and on-target adverse events related to pharmacological activity and may also lower the antigenic protein load per treatment and, thus, annually [31]. BoNTAs access neurons through a dual receptor mechanism. Initially, the neurotoxin is held close to the neuron by a weak interaction with negatively charged cell surface gangliosides. Vesicle fusion during acetylcholine release at the presynaptic membrane exposes the vesicle-resident synaptic vesicle glycoprotein-2 (SV2) receptor, allowing the neurotoxin to bind with high avidity and then undergo receptor-mediated endocytosis [32,33]. For the RTP004-containing DAXI formulation, RTP004 binds noncovalently to the neurotoxin, giving it a strong net positive charge, which has been shown to increase binding to negatively charged neuronal membranes compared to BoNTA with HSA [20]. This prolonged binding likely facilitates the increased uptake of DAXI compared with conventional BoNTAs, where the weak binding likely results in neurotoxin clearance soon after the injection [30]. This proposed increased uptake was shown in a study where increasing concentrations of RTP004, but not HSA, produced a dose-dependent increase in SNAP-25 cleavage in cells exposed to constant concentrations of BoNTA [21]. In addition, local diffusion of the neurotoxin is limited, most likely because of the interaction between the RTP004–neurotoxin molecule complex and negatively charged extracellular matrix elements in the injected area [19]. This is supported by data from a rodent model, in which the peptide was demonstrated to reduce post-injection diffusion [28]. Therefore, it is likely that, in addition to lower amounts of core neurotoxin administered per treatment, the lower incidence of adverse events with DAXI is also a result of reduced diffusion of the neurotoxin and limited exposure in non-target tissues. The lower rates of key adverse events with comparable amounts of core neurotoxin suggest that the titration of both the dose and retreatment interval may be performed with a wide range of dosing and without significantly increasing the risk of adverse events. Less core neurotoxin injected, at potentially fewer intervals, may also reduce the potential for the development of neutralizing antibodies [34].

A key strength of this analysis is that safety data were sourced from the pivotal registration studies and PI for each BoNTA product, an approach that has previously been used in this class of medications [23]. A limitation of the present analysis is that the selection of treated muscles and the distribution of doses across the selected muscles were at the discretion of the treating physician in each of the pivotal trials. Additionally, the PIs for the respective drugs in this class do not delineate adverse event rates by the muscle(s) injected. However, the use of safety data from pivotal trials overcomes the limitations associated with direct comparisons between single studies in clinical practice that contain considerably greater heterogeneity in patient selection, dose, and injected muscles. The use of safety data from the PI, which are collected during the pivotal Phase 3 clinical trials, allows for a more consistent comparison of neurotoxin dosing because of the substantially similar inclusion and exclusion criteria and the systematic and standardized manner in which adverse events are required to be collected in Phase 3 trials. In addition, the use of the data in PIs helps control for comparisons between BoNTAs that have been approved for different lengths of time. Lastly, it should be noted that the strong correlation between the core neurotoxin content and key adverse event rates documented in this analysis should not be extrapolated to indications beyond CD. Future analyses should venture to substantiate these findings across other indications.

## 4. Conclusions

In this analysis, we have described, for the first time, a strong association between the core neurotoxin amount (in nanograms) and the rates of key adverse events for BoNTAs in the treatment of CD. We have shown that using nanograms of the core neurotoxin is an effective method for comparing the safety of BoNTA products while also establishing the importance of formulation in the safety profile of this drug class. Using this method, we have shown that DAXI has a potentially wider safety margin compared with other approved BoNTA products for CD. This improved safety profile may be attributed to the peptide-enhanced bioavailability of the DAXI formulation, which enables lower core neurotoxin content to be administered, and reduced diffusion as a result of greater anchoring of the core neurotoxin to the injection site.

## 5. Materials and Methods

To assess the relationship between key adverse events and neurotoxin content for the four BoNTAs approved for CD [13,14,15,17], the approved doses from the units of activity were converted to the published core neurotoxin amounts for those doses (Table 1). These core neurotoxin amounts (in ng) were then plotted against the reported incidence of dysphagia and muscle weakness in each PI. The coefficients of determination (R^2^) and trendlines for the three conventional BoNTAs were estimated via a regression-based line of best fit by plotting event rates on the y-axis and core neurotoxin amounts on the x-axis.

Data were retrieved from the pivotal Phase 3 clinical trials that were used for the FDA approval for each product (Table 2), reported in each PI. All trials evaluated the effect of a single BoNTA treatment. For incobotulinumtoxinA 120 U and 240 U, the incidences of dysphagia and muscle weakness reported in the PI [15] are based on a pivotal, double-blind, placebo-controlled study [36]. For abobotulinumtoxinA 250 U, 500 U, and 1000 U, the incidences of dysphagia and muscle weakness reported in the PI [14] are based on a fixed-dose, double-blind, placebo-controlled trial of abobotulinumtoxinA [37] and an additional data point for the 500 U dose that was pooled from the double-blind, placebo-controlled phases of several other studies [38,39,40,41]. For onabotulinumtoxinA 236 U, the incidences of dysphagia and muscle weakness were derived from a double-blind, active-controlled study [42]; dysphagia was reported in the PI [13], and muscle weakness was reported in the response to the FDA clinical review [43]. For DAXI 125 U and 250 U, the incidences of dysphagia and muscle weakness reported in the PI [17] are based on a pivotal, double-blind, placebo-controlled study [22].

Treated muscles in cervical dystonia include the levator scapulae, longissimus, splenius capitis, splenius cervices, sternocleidomastoid muscle, and trapezoid muscle. Muscle selection is based on the patient’s head and neck position, localization of pain, muscle hypertrophy, patient response, and adverse event history, and lower doses are recommended for injections to the sternocleidomastoid muscle to minimize the occurrence of dysphagia (Table 3).

## Figures and Tables

**Table 1:** Core neurotoxin amounts for each BoNTA approved for cervical dystonia in the United States.

BoNTA	Brand Name	Approved Dose	Core Neurotoxin Amount
AbobotulinumtoxinA [14,35]	Dysport^®^, Ipsen Biopharm Ltd., Wrexham, UK	500 U 1000 U	2.7 ng 5.4 ng
IncobotulinumtoxinA [15,35]	Xeomin^®^, Merz Pharmaceuticals GmbH, Frankfurt, Germany	120 U 240 U	0.48 ng 0.96 ng
OnabotulinumtoxinA [13,35]	Botox^®^, AbbVie, Dublin, Ireland	236 U	2.13 ng
DaxibotulinumtoxinA [17]	DAXXIFY^®^, Revance Therapeutics, Inc., Nashville, TN, USA	125 U 250 U	0.56 ng 1.13 ng

Abbreviation: BoNTA—botulinum toxin A.

**Table 2:** A summary of the clinical trials included in the analysis of PI safety data.

BoNTA	Study Design	No. Randomized	Baseline Characteristics of BoNTA Treatment Arm	Incidence of Adverse Events Reported in Each PI	
				Dysphagia	Muscle Weakness
OnabotulinumtoxinA	Randomized, double-blind, active-controlled Duration: 20 weeks Comella et al., 2005 [42]	OnabotulinumtoxinA: 74 BoNTB: 65	Female: 57% Mean (SD) age: 57.0 y (11.8) CD duration: 8.1 y (6.4) CD severity ^a^: 18.9 (2.8) Previous BoNT treatment: 100%	236 U ^d^: 19%	236 U ^d^: 9%
AbobotulinumtoxinA	Randomized, double-blind, PBO-controlled Duration: 8 weeks Poewe et al., 1998 [37]	250 U: 19 500 U: 18 1000 U: 18 PBO: 20	Female: 48% Mean (SD) age: 47.0 y (11.5) ^b^ CD duration: 7.4 y (6.7) ^b^ CD severity ^c^: 250 U 14.3, 500 U 13.1, 1000 U 14.5 Previous BoNT treatment: 0%	250 U: 21% 500 U: 29% 1000 U: 39%	250 U: 11% 500 U: 12% 1000 U: 56%
AbobotulinumtoxinA	Randomized, double-blind, PBO-controlled Duration: 16 weeks Wissel et al., 2001 [41]	500 U: 35 PBO: 33	Female: 45.7% Mean (SD) age: 45.8 y (13.2) CD duration: 6.5 y (8.0) CD severity ^c^: 11.1 (1.7) Previous BoNT treatment: 68.6%	Pooled 500 U: 15% ^e^	Pooled 500 U: 16% ^e^
	Randomized, double-blind, PBO-controlled Duration: 20 weeks Truong et al., 2005 [40]	500 U: 37 PBO: 43	Female: 62.0% Mean (SD) age: 53.4 y (11.6) CD duration: 7.1 y (7.1) CD severity ^a^: 19.7 (2.6) Previous BoNT treatment: 75.7%		
	Randomized, double-blind, PBO-controlled Duration: 12 weeks Truong et al., 2010 [39]	500 U: 55 PBO: 61	Female: 67.0% Mean (SD) age: 51.9 y (13.4) CD duration: 12.0 y (8.8) CD severity ^a^: 20.4 (3.0) Previous BoNT treatment: 82.0%		
	Randomized, double-blind, PBO-controlled Duration: 12 weeks Lew et al., 2018 [38]	500 U: 89 PBO: 45	Female: 66.3% Mean (SD) age: 57.3 y (11.1) CD duration: NR CD severity ^a^: NR Previous BoNT treatment: 64.0%		
IncobotulinumtoxinA	Randomized, double-blind, PBO-controlled Duration: 20 weeks Comella et al., 2011 [36]	120 U: 78 240 U: 81 PBO: 74	Female: 120 U 51%, 240 U 54% Mean (SD) age: 120 U 52.8 y (11.5), 240 U 53.2 y (12.2) CD duration: 120 U 9.3 y (8.4), 240 U 9.7 y (9.0) CD severity ^a^: 120 U 18.0 (4.4), 240 U 18.6 (4.1) Previous BoNT treatment: 120 U 60.3%, 240 U 61.4%	120 U: 13% 240 U: 18%	120 U: 7% 240 U: 11%
DaxibotulinumtoxinA (DAXI)	Randomized, double-blind, PBO-controlled Duration: 36 weeks Comella et al., 2024 [22]	125 U: 125 250 U: 130 PBO: 46	Female: 125 U 69.6%, 250 U 58.6% Mean (SD) age: 125 U 57.2 y (13.4), 250 U 58.6 y (10.6) CD duration: 125 U 10.8 y (8.8), 250 U 10.5 y (9.6) CD severity: NR Previous BoNT treatment: 120 U 88.0%, 240 U 85.4%	125 U: 2% 250 U: 4%	125 U: 5% 250 U: 2%

^a^ Toronto Western Spasmodic Torticollis Rating Scale severity score. ^b^ Across all groups. ^c^ Tsui scale. ^d^ Mean dose administered, reported in PI. ^e^ Pooled data from multiple studies. Abbreviations: BoNT—botulinum toxin; CD—cervical dystonia; NR—not reported; PBO—placebo; PI—prescribing information; SD—standard deviation; y—year.

**Table 3:** Summary of muscle selection and dosing from clinical trials and PI for each BoNT.

BoNT Dosage Units Study	Muscle Selection and Dosing in Pivotal Clinical Trials	Muscle Selection and Dosing Recommendations in PI
OnabotulinumtoxinA 236 U Comella et al., 2005 [42]	Muscle selection, dosing, injection sites, and the use of EMG were at the discretion of the injecting physician	Dosing is based on the patient’s head and neck position, localization of pain, muscle hypertrophy, patient response, and adverse event history Use a lower initial dose (100 U) in the SM in treatment-naïve patients to minimize the occurrence of dysphagia Dilution is 200 U/2 mL, 200 U/4 mL, 100 U/1 mL, or 100 U/2 mL depending on volume and number of injection sites desired to achieve treatment objectives No more than 50 U per site
AbobotulinumtoxinA 250 U 500 U 1000 U Poewe et al., 1998 [37]	0.75 mL was divided into two injection sites in the upper one third of the SM contralateral to the direction of chin rotation (0 U, 75 U, 150 U, 300 U) 1.75 mL was divided into two injection sites in the SP (0 U, 175 U, 350 U, 700 U) ipsilateral to the direction of head turn Injections to neck muscle were not guided by EMG	Initial dose is 500 U, divided among the affected muscles Use a lower dose in the SM to reduce the occurrence of dysphagia Total dose administered in a single treatment should be between 250 U and 1000 U Titrate in 250 U steps according to subject’s response Dilution is 500 U/1 mL, 500 U/2 mL, 300 U/0.6 mL
AbobotulinumtoxinA 500 U Wissel et al., 2001 [41]	Muscles injected: two or three of the following: SM, SP, TP, and LS from either side Muscle selection: based on clinical assessment (direction of head deviation and/or shoulder elevation, visible hypertrophy, palpable stiffness, and pain localization) 1.0 mL (500 U) was divided into two injection sites as follows: SM 0.2–0.4 mL (100–200 U), SP 0.5–0.7 mL (250–350 U), TP 0.2–0.4 mL (100–200 U), LS 0.2–0.4 mL (100–200 U) Exact dose per muscle was determined within these guidelines at the discretion of the injecting physician	
AbobotulinumtoxinA 500 U Truong et al., 2005 [40]	Muscle selection included two, three, or four clinically indicated neck muscles in a single dosing session, with or without EMG Dosing and number of injection sites were at the discretion of the injecting physician	
AbobotulinumtoxinA 500 U Truong et al., 2010 [39]	Muscle selection included two, three, or four clinically indicated neck muscles in a single dosing session, with or without EMG Dosing and number of injection sites were at the discretion of the injecting physician Patients received a minimum of 250 U or a maximum of 1000 U per treatment cycle	
AbobotulinumtoxinA 500 U Lew et al., 2018 [38]	Dosing included 500 U vial/2 mL in a minimum of two clinically affected neck muscles; injection to the SM was limited to 0.6 mL (150 U), to reduce the occurrence of dysphagia, per the PI Use of EMG was at the discretion of the injecting physician	
IncobotulinumtoxinA 120 U, 240 U Comella et al., 2011 [36]	Dosing included either 120 U or 240 U of incobotulinumtoxinA in a single treatment. The total volume administered was 4.8 mL for the injection The number of injection sites per muscle, the volume injected, and the use of EMG were at the discretion of the injecting physician	Dosing is based on previous treatment history (past dose, response, duration of effect, adverse events) The recommended initial total dose is 120 U Muscles usually injected include the SM, LS, SP, scalenus, and/or the TP The dose and number of injection sites per muscle should be individualized based on the number and location of the muscle(s) to be treated, the degree of spasticity/dystonia, muscle mass, bodyweight, and response to any previous neurotoxin injections
DaxibotulinumtoxinA (DAXI) 125 U, 250 U Comella et al., 2024 [22]	Muscles injected: two or three of the SM, LS, longissimus, scalenus complex, SP, splenius cervices, and TP, from either side Muscle selection: based on clinical assessment (the position of the head, neck, and shoulders, muscle hypertrophy, and pain localization) 2.5 mL (250 U) was divided into the injection sites as follows: SM 0.2–0.5 mL (20–50 U), LS or longissimus 0.2–0.6 mL (20–60 U), scalenus complex 0.2–0.3 mL (20–30 U), SC or splenius cervices 0.2–1.0 mL (20–100 U), TP 0.3–0.8 mL (30–80 U) 2.5 mL (125 U) was divided into the injection sites as follows: SM 0.2–0.5 mL (10–25 U), LS or longissimus 0.2–0.6 mL (10–30 U), scalenus complex 0.2–0.3 mL (10–15 U), SP or splenius cervices 0.2–1.0 mL (10–50 U), TP 0.3–0.8 mL (15–40 U) The total volume was required to be injected Use of EMG or other imaging modalities for guiding injections was at the discretion of the injecting physician	Recommended dose (125 U or 250 U) is to be divided among affected muscles The initial dose is based on previous treatment history (past dose, response, duration of effect, adverse events) Use a lower dose in the SM to reduce the occurrence of dysphagia Dilution is 100 U/1 mL or 100 U/2 mL

Abbreviations: BoNT—botulinum toxin; CD—cervical dystonia; EMG—electromyography; LS—levator scapulae muscle; SP—splenius capitis muscle; SM—sternocleidomastoid muscle; TP—trapezoid muscle.

## Data Availability

The original contributions presented in the study are included in the article; further inquiries can be directed to the corresponding author.
